# Supplementary figures and images for: A Preliminary Study on the Characteristics of microRNAs in Ovarian Stroma and Follicles of Chuanzhong Black Goat during Estrus
Source: Genes (Basel). 2020 Aug 21;11(9):970. doi: 10.3390/genes11090970 (PMC7564575; doi:10.3390/genes11090970)

# PCA

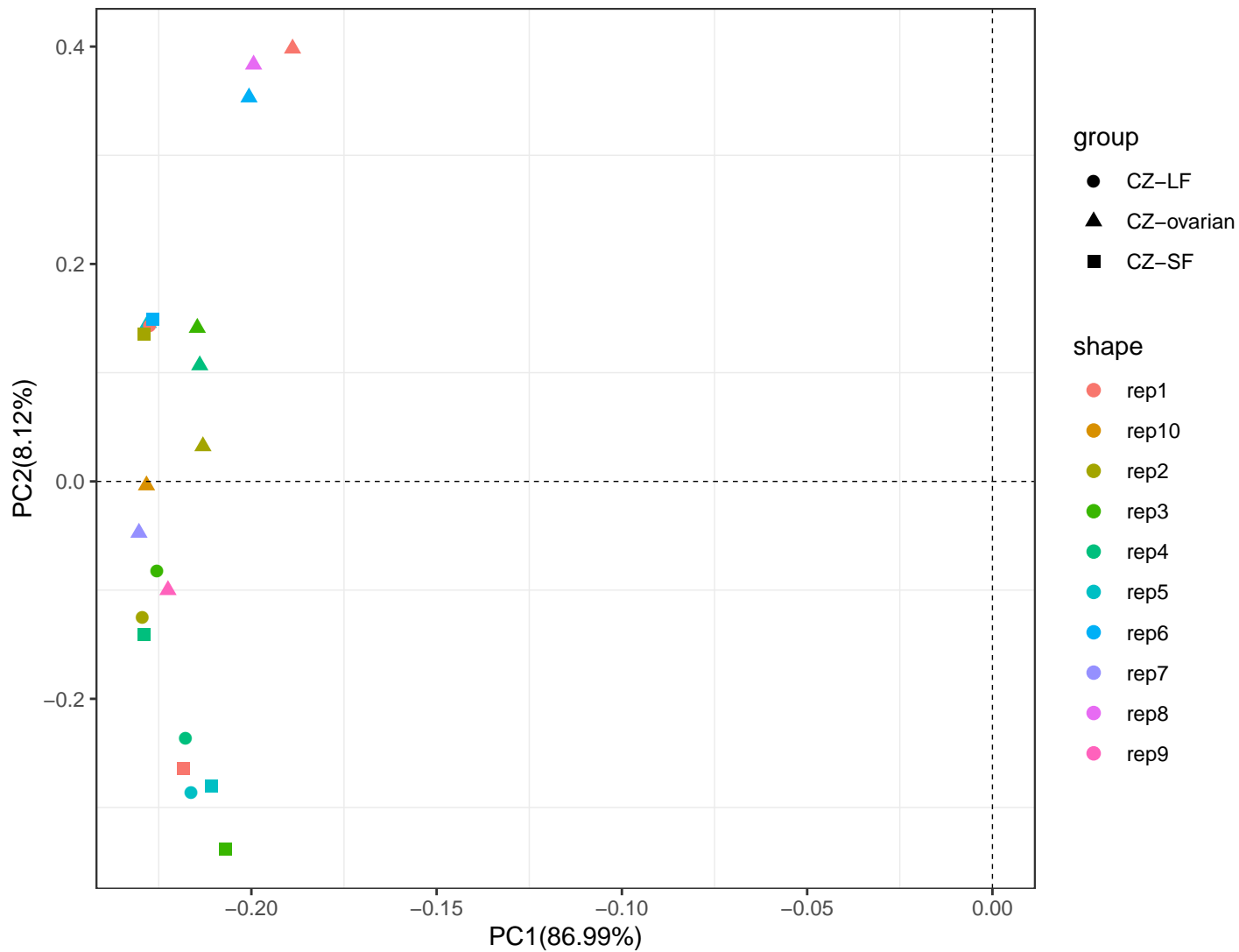

Supplement: Supplementary file 1 [file genes-11-00970-s001.zip › Figure S1.pdf]
